# Association of COVID-19 infection and the risk of new incident diabetes: a systematic review and meta-analysis

**DOI:** 10.3389/fendo.2024.1429848

**Published:** 2024-08-26

**Authors:** Jingye Zhou, Yuzhu Wang, Ruolan Xu

**Affiliations:** ^1^ International Medical College, Chongqing Medical University, Chongqing, China; ^2^ College of Life Sciences, University of Leicester, Leicester, United Kingdom

**Keywords:** COVID-19, long COVID, SARS-CoV-2, diabetes mellitus, public health

## Abstract

**Background:**

As the world population recovers from the COVID-19 infection, a series of acute sequelae emerge including new incident diabetes. However, the association between COVID-19 infection and new incident diabetes is not fully understood. We purpose to determine the risk of new incident diabetes after COVID-19 infection.

**Methods:**

PubMed, Embase, and Cochrane Library were used as databases to search for cohort studies published from database inception to February 4, 2024. Two reviewers independently conducted the study screening, data extraction, and risk of bias assessment. A random-effects model was adopted to pool the hazard ratio (HR) with corresponding 95% confidence intervals (CI). Subgroup analysis was conducted to explore the potential influencing factors.

**Results:**

A total of 20 cohort studies with over 60 million individuals were included. The pooling analysis illustrates the association between COVID-19 infection and an increased risk of new incident diabetes (HR = 1.46; 95% CI: 1.38-1.55). In subgroup analysis, the risk of type 1 diabetes was HR=1.44 (95% CI: 1.13-1.82), and type 2 diabetes was HR=1.47 (95% CI: 1.36-1.59). A slightly higher risk of diabetes was found in males (HR=1.37; 95% CI: 1.30-1.45) than in females (HR=1.29; 95% CI: 1.22-1.365). The risk of incident diabetes is associated with hospitalization: non-hospitalized patients have an HR of 1.16 (95% CI: 1.07-1.26), normal hospitalized patients have an HR of 2.15 (95% CI: 1.33-3.49), and patients receiving intensive care have the highest HR of 2.88 (95% CI: 1.73-4.79).

**Conclusions:**

COVID-19 infection is associated with an elevated risk of new incident diabetes. Patients ever infected with COVID-19 should be recognized as a high-risk population with diabetes.

**Systematic review registration:**

https://www.crd.york.ac.uk/prospero, identifier CRD42024522050.

## 1 Introduction

Diabetes is a chronic non-communicable disease characterized by impaired glucose metabolism that results in persistently raised blood glucose in the context of insufficient insulin caused by autoimmune-mediated destruction of pancreatic β-cells or insulin resistance combined with pancreatic β-cell insufficiency (1). Despite significant process has been made in the exploration of risk factors for diabetes and the implementation of prevention programs, there is a globally increasing incidence and prevalence of the disease (2). Early detection and intensive patient-centered management are expected to optimize the prognosis, reducing morbidity and mortality by preventing or delaying complications (2). A previous study has explored the primary risk factors of diabetes, including BMI, genetics, atmosphere, diet habit, drug use, sedentary way of life, lack of physical exercise, smoking, alcoholic beverages, dyslipidemia, hyperinsulinemia, and improved glucagon activity (3). Recently, the bidirectional interaction between coronavirus disease 2019 (COVID-19) and diabetes has been revealed (4–7). COVID-19 presumably increases the risk of new incident diabetes (8, 9).

The pandemic of COVID-19 caused by severe acute respiratory syndrome coronavirus 2 (SARS-CoV-2) is recognized as the greatest worldwide public health threat of this century (10). Although the World Health Organization (11) has declared that COVID-19 is no longer a public health emergency of international concern in May 2023, it continues to circulate and evolve, and remains a potentially serious risk to public health. Simultaneously, sequelae after the acute phase of COVID-19 (called long COVID) have aroused wild attention in the medical field (12). Patients with long COVID experience lingering symptoms across multiple organ systems, with common new incident conditions such as diabetes (13). Current reviews revealed an association between COVID and increased incidence of diabetes (14–16), but Zareini et al. (17) indicated an opposite perspective. Therefore, we systematically reviewed the existing cohort studies to clarify the association between COVID-19 and the risk of new incident diabetes.

## 2 Methods

This systematic review and meta-analysis were conducted in accordance with the Preferred Reporting Items for Systematic Review and Meta-analysis (PRISMA) guidelines (18). The study protocol was registered in the International Prospective Register of Systematic Reviews (PROSPERO) platform on March 12, 2024 (CRD42024522050).

### 2.1 Search strategy

We systematically searched PubMed, Embase, and Cochrane Library for studies published up to February 4, 2024. No language restrictions were applied, and the search strategy combined the use of medical subject headings (MeSH) and free text. The search terms were related to COVID-19, Post-Acute COVID-19 Syndrome, Diabetes Mellitus, and risk. The full search strategies are included in **Supplementary Tables 1**-**3**. The reference lists of other published meta-analyses were also considered to identify relevant cohort studies.

### 2.2 Eligibility criteria

Original research studies must meet all the following criteria to be included: (1) the study design was a prospective or retrospective cohort study investigating the association between COVID-19 and the risk of new incident all-type diabetes (no prior history of diabetes); (2) COVID-19 and diabetes were defined based on medical records or International Classification of Diseases (ICD) codes; (3) the hazard ratio (HR) or odds ratio (OR) and its corresponding 95% confidence interval (CI) were reported.

The following were excluded: reviews, study protocols, and commentaries.

### 2.3 Study selection

Study selection was performed by two reviewers (JYZ and YZW), independently. Titles and abstracts were first screened to exclude duplicate and irrelevant articles. Thereafter, the full texts were examined to identify all eligible studies. If multiple studies conducted assessments from the same database, we include the one with more adequate data based on its sample size and follow-up duration. Any disagreements were resolved by discussing them with the third reviewer (RLX).

### 2.4 Data extraction

Two reviewers mentioned above (JYZ and YZW) extracted data independently consulting the guidelines on data extraction for systematic reviews and meta-analysis (19). Predesigned forms were used for data extraction, including the first author, year of publication, country, study type, data source, sample size, follow-up duration, mean age, diagnosis criteria of COVID/diabetes, type of diabetes, interval (interval between the first diagnosis of COVID and the onset of diabetes). Disagreements were resolved by consensus with all researchers (JYZ, YZW, and RLX).

### 2.5 Risk of bias

The quality of the included studies was assessed using the Newcastle-Ottawa scale (NOS) (20). A “star system” was used to judge the studies from three broad perspectives: the selection of participants a measurement of exposure, the comparability of the study groups, and the assessment of outcomes and adequacy of follow-up. Each assessment was carried out by two reviewers (JYZ and YZW) separately and repeatedly. Disagreements were solved by discussion with the third reviewer (RLX).

### 2.6 Statistical analysis

For this meta-analysis, we sought to identify HRs and 95% CI to assess the association between COVID-19 and the risk of new incident diabetes. Heterogeneity among the studies was evaluated by the χ^2^ -test and the I^2^ -values. If I^2^ > 50%, a random-effects model of analysis was used. We applied a sensitivity analysis by excluding one study each time and rerunning it to verify the robustness of the overall effects. The funnel plot was constructed to inspect and visualize publication bias, and Egger’s regression test was conducted to statically assess it. Subgroup analyses were performed if two or more cohorts were identified. p-values < 0.05 were considered to be statistically significant. All analyses were performed using Stata software (Stata Corp V.14, Texas, USA).

## 3 Results

### 3.1 Literature search

**Figure 1:**
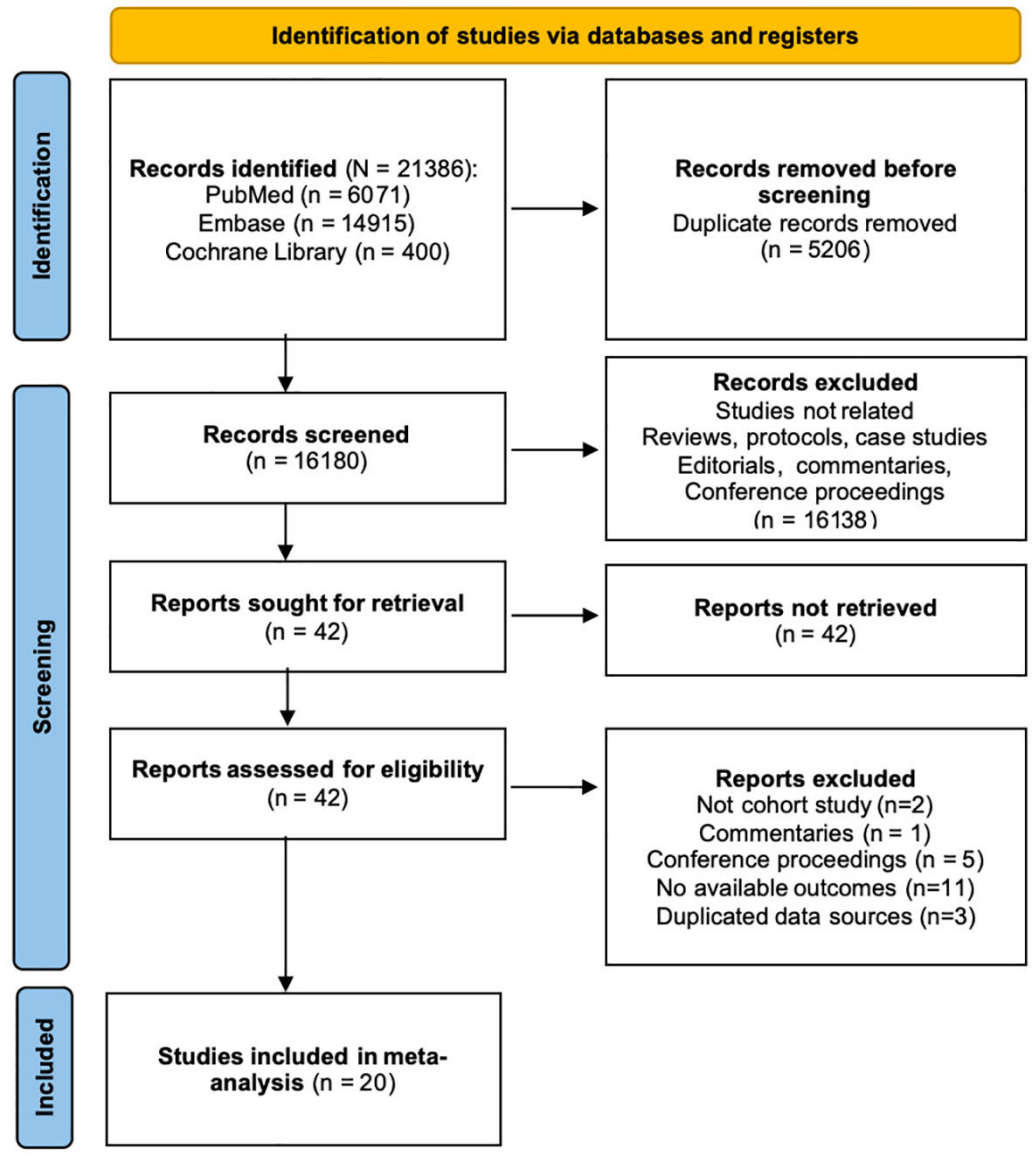
Studies screening flow diagram.

21386 results were obtained after the systematic search. After removing duplicate content and screening the title and abstract, 42 articles were potentially eligible. Full-text articles were all accessible in the remaining 42 studies. Twenty-two studies were excluded after full-text review: 2 were not cohort studies, 1 was commentary, 5 were conference proceedings, 12 did not provide our interested effect sizes and 3 used duplicated data sources. Bowe et al. (21) used the same dataset as Xie and Al-Aly (22) but focused on different outcomes, so we included both. 20 cohort studies (17, 21–39) were included in the meta-analysis. The PRISMA flow diagram illustrating the search and selection process is provided in **Figure 1**.

### 3.2 Study characteristics

This meta-analysis included 20 cohort studies covering 60,221,176 individuals, which were published between 2021 and 2023. Out of the 20 studies, one was a prospective cohort study, while the other 19 were retrospective studies. Among all the studies included, one reported gestational diabetes mellitus (GDM), five reported type 1 diabetes (T1D), seven reported type 2 diabetes (T2D), and eight reported both T1D and T2D. The follow-up duration of participants ranges from 3 to 84 months. Additional characteristics of the included studies are shown in **Table 1**.

**Table 1:** Basic characteristics of the included studies.

Author	Year	Country	Study type	Data source	Sample size, n	Follow-up duration, months	Mean age, years	Diagnosis of COVID-19/diabetes	Diabetes type	Interval, days	NOS scores	Adjusted factors
Aslam et al. (23)	2023	Pakistan	Retrospective cohort	Department of respiratory physiology and medicine of independent University Hospital	COVID: 55 Control: 50	5	COVID: 39.5 Control: 25.65	COVID: medical records, RT-PCR Diabetes: medical records, HbA1c	T2D	>1	7	NR
Barrett et al. (24)[1]	2022	USA	Retrospective cohort	IQVIA	COVID: 80,893 Control: 404,465	12	COVID: 12.3 Control: 12.3	COVID: medical records, ICD-10 Diabetes: medical records, ICD-10	T1D/T2D	>30	8	Age, sex
Barrett et al. (24)[2]	2022	USA	Retrospective cohort	Health Verity	COVID: 439,439 Control: 439,439	16	COVID: 12.7 Control: 12.7	COVID: medical records, PCR, ICD-10 Diabetes: medical records, ICD-10	T1D/T2D	>30	8	Age, sex
Bowe et al. (21)	2022	USA	Retrospective cohort	VHSUS Veterans Health Administration	COVID: 234,990 Control: 5,334,729	27.8	COVID: 60.12 Control: 60.12	COVID: medical records, RT-PCR, ICD-10 Diabetes: medical records, HbA1c, ICD-10	T2D	>1	6	Age, sex, race, BMI, vaccination status, area deprivation index, smoking
Choi et al. (25)	2023	South Korea	Retrospective cohort	Health Insurance Review and Assessment Service	COVID: 348,180 Control: 1,044,540	12.5	COVID: 43.3 Control: 43.3	COVID: medical records, ICD-10 Diabetes: medical records, ICD-10	T2D	>30	9	Age, sex, hypertension, dyslipidemia
Daugherty et al. (26)	2021	USA	Retrospective cohort	United Health Group Clinical Discovery Database	COVID: 266,586 Control: 8,980,919	10	COVID: 41.7 Control: 42.4	COVID: medical records, PCR, ICD-10 Diabetes: medical records, ICD-10	T2D	>21	7	Age, sex, race
Jun Zhang et al. (27)	2022	China	Prospective cohort	Wuhan Hospital of Traditional Chinese Medicine	COVID: 171 Control: 77	3.3	Total: 61.0	COVID: medical records, RT-PCR Diabetes: fasting blood glucose	T1D/T2D	After discharge	7	Age, sex, BMI, smoking, drinking, hypertension, comorbidities
Kendall et al. (28)	2022	USA	Retrospective cohort	TriNetX	COVID: 314,917 Control: 776,577	6	COVID: 10.3 Control: 10.3	COVID: medical records, ICD-10 Diabetes: NR	T1D	>1	7	NR
Kwan et al. (29)	2023	USA	Retrospective cohort	Cedars-Sinai Health System	Total: 23,709	6	Total: 47.4	COVID: medical records, ICD-9, ICD-10 Diabetes: medical records, ICD-9, ICD-10	T1D/T2D	0-90	6	Age, sex, hypertension, hyperlipidaemia
Lu et al. (30)	2023	USA	Retrospective cohort	Montefiore Health System in Bron	COVID: 19,427 Control: 5,730	3	NR	COVID: medical records, RT-PCR Diabetes: medical records, ICD-10	T2D	>1	9	Age, sex, race, ethnicity, BMI, hypertension, HF, CKD, COPD
McKeigue et al. (31)	2022	UK	Retrospective cohort	REACT-SCOT	COVID: 365,080 Control: 1,484,331	20.6	Range: <35	COVID: medical records, PCR Diabetes: medical records	T1D	>1	9	Sex, vaccination status
Naveed et al. (32)	2023	Canada	Retrospective cohort	British Columbia COVID-19 Cohort	COVID: 125.987 Control: 503,948	24	COVID: 45 Control: 41.5	COVID: medical records, RT-PCR Diabetes: medical records, ICD-9, ICD-10	T1D/T2D	>30	7	Age, sex, vaccination status
Noorzae et al. (33)	2023	Denmark	Retrospective cohort	The Danish Civil Registration System	COVID: 419,260 Control: 1,174,677	30	Range: 0-17	COVID: medical records Diabetes: medical records, ICD-10	T1D	>30	7	Age, sex, comorbidities, vaccination status
Qeadan et al. (34)	2022	USA	Retrospective cohort	Cerner Real-World Data	COVID: 2,489,266 Control: 24,803,613	22	COVID: 44.5 Control: 41.1	COVID: medical records, laboratory test Diabetes: medical records, ICD-10	T1D	>1	6	Age, sex, race, ethnicity, marital status, region
Rathmann et al. (35)	2022	Germany	Retrospective cohort	Disease Analyzer	COVID: 35,865 Control: 35,865	16.7	COVID: 42.6 Control: 42.6	COVID: medical records, ICD-10 Diabetes: medical records, ICD-10	T2D	>1	9	Age, sex, obesity, hypertension, hyperlipidemia
Rege et al. (36)	2023	Israel	Retrospective cohort	Clalit	COVID: 157,936 Control: 157,936	10.9	COVID: 43 Control: 43	COVID: medical records, PCR Diabetes: medical records, ICD-9	T1D/T2D	>1	9	Age, BMI, socioeconomic status, hypertension, dyslipidemia, smoking status
Rezel-Potts et al. (37)	2022	UK	Retrospective cohort	CPRD Aurum	COVID: 428,650 Control: 428,650	12	COVID: 35 Control: 35	COVID: medical records, PCR Diabetes: medical records, HbA1c	T1D/T2D	0-120	9	Age, sex, ethnicity, BMI, smoking status
Soysal and Yilmaz (38)	2022	Turkey	Retrospective cohort	Obstetrics outpatient clinic of the Obstetrics and Gynecology Department of Ankara	COVID: 150 Control: 150	27	COVID: 28.73 Control: 28.29	COVID: medical records Diabetes: medical records, OGTT	GDM	>1	9	NR
Xie and Al-Aly (22)	2022	USA	Retrospective cohort	VHA	COVID: 181,280 Control: 4,278,701	11.6	COVID: 60.9 Control: 61.5	COVID: medical records, laboratory test Diabetes: medical records, ICD-10	T2D	>30	6	Age, sex, race, area deprivation index, BMI, smoking status, comorbidities
Yongkang Zhang et al. (39)	2022	USA	Retrospective cohort	PCORnet	COVID: 316,249 Control: 2,775,331	3	Non-hospitalized COVID: 48.9 Hospitalized COVID: 59.9 Non-hospitalized control: 52.8 Hospitalized control: 55.9	COVID: medical records, PCR, lCD-10 Diabetes: medical records, ICD-10	T1D/T2D	31-150	6	Age, sex, race, BMI, ethnicity, smoking status, comorbidities
Zareini et al. (17)	2023	Denmark	Retrospective cohort	Danish National Patient Registry; Danish National Prescription Registry; The Danish Cause of Death Registry; Danish Population Registry Danish Microbiology Database	COVID: 338,670 Control: 1,004,688	84	Total: 12.8	COVID: medical records, PCR Diabetes: medical records	T1D	>1	9	Age, sex, vaccination status

T1D, type 1 diabetes; T2D, type 2 diabetes; GDM, gestational diabetes mellitus; PCR, polymerase chain reaction; RT-PCR, reverse transcription polymerase chain reaction; ICD-10, International Classification of Diseases 10th Revision; ICD-9, International Classification of Diseases 9th Revision; BMI, body mass index; OGTT, oral glucose tolerance test; HF, heart failure; CKD, chronic kidney diseases; COPD, chronic obstructive pulmonary disease; NR, not reported.

### 3.3 Quality assessment

According to the NOS criteria, the average score of all included cohort studies was 8, and the score for five trials (21, 22, 29, 34, 39) was 6 while other 14 trials (17, 23–28, 30–33, 35–38) was 7 or above, indicating that all cohort studies were of relatively high quality in this meta-analysis. The score of each study is shown in **Table 1**.

### 3.4 COVID-19 infection and the risk of overall diabetes

**Figure 2:**
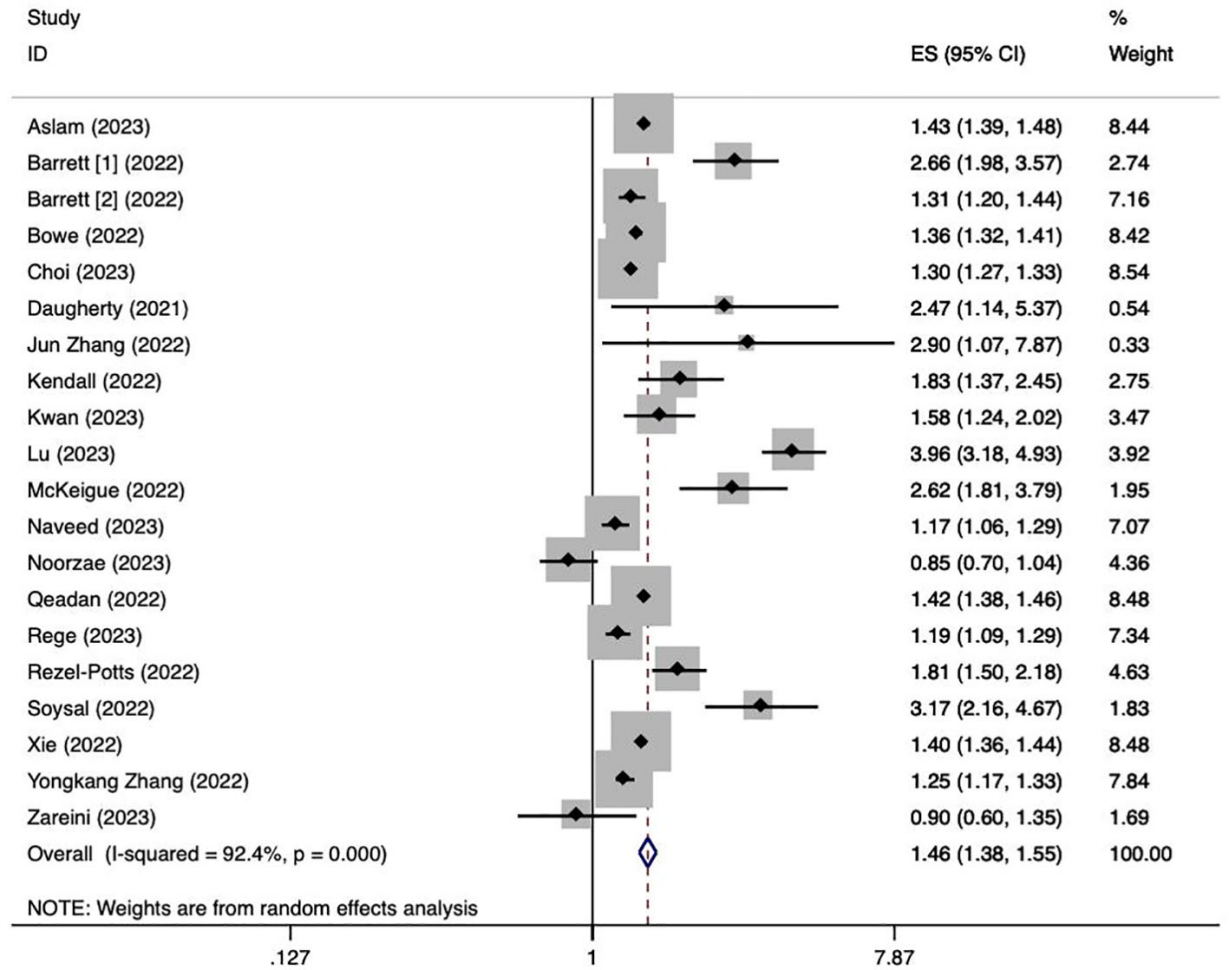
Meta-analysis of the risk of overall diabetes after a history of COVID-19. Barrett [1]: data from IQVIA; Barrett [2]: data from Health Verity.

We used data from twenty cohort studies (17, 21–39) to explore the association between a history of COVID-19 and the risk of overall diabetes. The pooling analysis reveals that a history of COVID-19 infection is associated with an increased risk of overall diabetes (HR = 1.46; 95% CI: 1.38-1.55; I^2^ = 92.4%, p < 0.001; **Figure 2**). The significant heterogeneity in the included studies was interpreted by using a random effect model meta-analysis. Sensitivity analysis shows that none of the individual studies reversed the pool-effect size, indicating that the results are robust (**Supplementary Figure 1**).

### 3.5 Subgroup analysis

The results of the subgroup analysis are summarized in **Table 2**. Increased risks of new incident T1D and T2D are associated with COVID-19 infection but there are no significant differences between the two, T1D (HR=1.44; 95% CI: 1.13-1.82; I^2^ = 89.1%, p = 0.003), and T2D (HR=1.47; 95% CI: 1.36-1.59; I^2^ = 94.6%, p < 0.001). With stratification by sex, males (HR=1.37; 95% CI: 1.30-1.45; I^2^ = 86.2%, p < 0.001) are observed with higher risks compared to those for females (HR=1.29; 95% CI: 1.22-1.365; I^2^ = 75.6%, p < 0.001). In hospitalization-stratified analysis, the pooled risks of new incident diabetes are significantly higher for patients in intensive care (HR=2.88; 95% CI: 1.73-4.79; I^2^ = 95.4%, p < 0.001) than those for non-hospitalized patients (HR=1.16; 95% CI: 1.07-1.26; I^2^ = 98.8%, p = 0.002) and normal hospitalized patients (HR=2.15; 95% CI: 1.33-3.49; I^2^ = 94.6%, p < 0.001). No significant associations are found in the stratification of vaccination status. For populations from different regions, pooled risks were evaluated as America (HR=1.52; 95% CI: 1.40-1.64; I^2^ = 93.0%, p < 0.001), Asian (HR=1.39; 95% CI: 1.25-1.54; I^2^ = 92.3%, p < 0.001), and Europe (HR=1.60; 95% CI: 1.03-2.49; I^2^ = 90.8%, p = 0.036).

**Table 2:** Subgroup analysis for the risk of diabetes in patients with COVID-19.

Subgroups	Included studies, n	HR (95% CI)	Heterogeneity	
			*I^2^* (%)	*p*-values
Type of diabetes				
Type 1 diabetes	6	1.44 (1.13,1.82)	89.1%	0.003
Type 2 diabetes	8	1.47 (1.36,1.59)	94.6%	0.000
Sex				
Male	7	1.37 (1.30,1.45)	86.2%	0.000
Female	7	1.29 (1.22,1.36)	75.6%	0.000
Hospitalization				
Non-hospitalized	3	1.16 (1.07,1.26)	87.5%	0.000
Hospitalized	4	2.15 (1.33,3.49)	98.8%	0.002
Intensive care	4	2.88 (1.73,4.79)	95.4%	0.000
Vaccination status				
0 vaccine received	4	1.27 (0.99,1.63)	86.3%	0.064
1 vaccine received	4	1.09 (0.73,1.63)	67.9%	0.676
≥2 vaccine received	3	1.21 (0.92,1.60)	55.7%	0.178
Region				
America	11	1.52 (1.40,1.64)	93.0%	0.000
Asian	5	1.39 (1.25,1.54)	92.3%	0.000
Europe	5	1.60 (1.03,2.49)	90.8%	0.036

### 3.6 Publication bias

**Figure 3:**
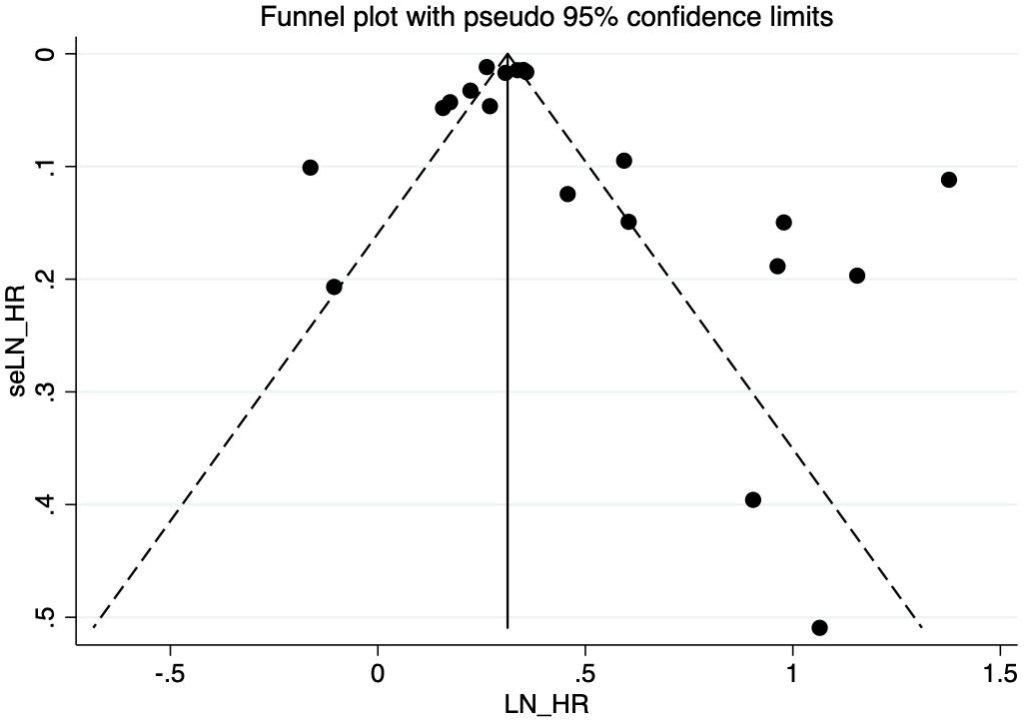
Publication bias of the risk of new incident diabetes caused by COVID-19 infection.

There is no evidence of a significant publication bias in the COVID-19 infection and risk of new incident diabetes revealed from the visual inspection of the funnel plot (**Figure 3**). Egger’s test (P = 0.166) shows no publication bias in our meta-analysis either.

## 4 Discussion

### 4.1 Main findings

We conducted a meta-analysis of 20 cohort studies covering 60,221,176 individuals, which provided a comprehensive evaluation of the association between COVID-19 and new incident diabetes. We find a significant increase in the risk of all-type diabetes among individuals after COVID-19 infection, with an overall 1.46-fold increase in risk. This indicated that COVID-19 infection might be an independent risk factor for new incident diabetes. The importance of screening, prevention, and management of diabetes for patients ever infected with COVID-19 should be emphasized.

### 4.2 Comparison with previous studies

Our analysis demonstrated a consistent result with previous reviews (14–16), showing that COVID-19 infection increased the risk of all-type diabetes. In addition, Li et al. (40) explored the relationship between new-onset diabetes, hyperglycemia, and COVID-19 infection, showing an elevated incidence and risk. In a review that specifically targeted T2D (41), a higher prevalence of diabetes in people with previous COVID-19 was illustrated, which further corroborated our findings. Compared to prior studies, we added more recent studies and analyzed the data in subgroups, to provide stronger evidence for the association between COVID-19 and diabetes. Simultaneously, we only included data from cases with a confirmed diagnosis of diabetes, contributing to reduced clinical heterogeneity and greater reliability. Although the risk variance between T1D and T2D is not significant in this analysis, a previous study found a higher risk for new incident T2D than T1D for all included cohorts (16). They also indicated that males with COVID-19 were associated with a higher risk of diabetes compared to females, which echoed our conclusions. We assessed the risks between subgroups of hospitalization, vaccination status, and incident diabetes for the first time, and found that an increased risk of diabetes was associated with the exacerbation of hospitalization. However, the risk of new incident diabetes in patients who received vaccination was not statistically significant.

### 4.3 Interpretation of findings

So far, the pathophysiological mechanism of the association between COVID-19 and diabetes is not entirely clear. It has been suggested that SARS-CoV-2 specifically induces the damage of β-cells, thereby impairing insulin production (42, 43). Angiotensin-converting enzyme 2 (ACE2) is the main receptor of SARS-CoV-2 to gain entry into human cells (44) Several studies have found the ACE2 expression in pancreatic β-cells (42, 45–47), leading to speculation that SARS-CoV-2 may triggers β-cell damage by penetrating the cells using ACE2 (48). In addition to ACE2, other SARS-CoV-2 related entry factors such as TMPRSS2, NRP1, and TRFC are also expressed in pancreatic β-cells, which might play roles in β-cell damage through similar mechanisms (42, 49). However, the expressions of ACE2 and TMPRSS2 in pancreatic β-cells were doubted in other studies (50–52). Therefore, further research is necessary.

ACE2 is a key enzyme in the renin-angiotensin system (RAS). Membrane-bound ACE2 is responsible for catalyzing the conversion from Ang II into Ang-(1-7) (53). Down-regulation of ACE2 is found in patients with COVID-19 that enhances activation of the RAS axis, resulting in decreased insulin and glucose delivery to tissues and impairment of insulin signaling pathways, all of which lead to insulin resistance (54, 55). Additionally, uncontrolled inflammatory response caused by RAS imbalance might account for the potential role in pancreatic dysfunction (53, 54).

Autopsy tissue from deceased COVID-19 patients showed that local inflammation and infiltration of immune cells were associated with impairment of β-cells, causing various degrees of metabolic dysregulation (50). SARS-CoV-2 triggers a macrophage-mediated cytokine storm in which the overactivation of immune cells and persistently increasing cytokines promote excessive inflammation and further induced β-cell damage (56). SARS-CoV-2 induce a decreased chromatin-modifying enzyme SETDB2, causing increased transcription of inflammatory cytokines which impair the pancreas (56).

Steroids are used to treat COVID-19, but their pharmacological effects pose extra burden on blood glucose control (57). Steroid-induced hyperglycemia in patients with COVID-19 may be associated with an increased risk of new incident diabetes (9). A cohort study revealed a higher risk of diabetes in COVID-19 patients using glucocorticoids compared to those without steroid treatments (58), which corroborates this perspective.

Lockdowns during the COVID-19 pandemic slowed the rate of infection but caused negative mental health consequences and adverse health-related behaviors, including reduced physical activities, unhealthy eating, smoking, and binge drinking, which are risk factors for diabetes (59). Symptoms of long COVID such as fatigue, muscle pain, and dyspnea, limit exercise capacity (60), therefore sedentary lifestyles have become common. These changes of lifestyle have a series of pathophysiological effects, including metabolic consequences represented by insulin resistance, which might increase the risks of new incident diabetes (61).

In the subgroup analysis, males with a history of COVID-19 have a higher risk of new incident diabetes than females. A previous study has shown that males infected with COVID-19 are more susceptible to worse outcomes and death, independent of age (62). From another perspective, a study on rats indicated a gender-related difference of ACE2 expression, that ACE2 content was slightly lower in males compared to females (63). This might be attributed to diabetes-related pathophysiological changes. Considering vaccination has shown a potential effectiveness on improvement in long-COVID symptoms (64), it might be also helpful to prevent new incident diabetes in patients ever infected with COVID-19, which accounted for the insignificant association between COVID-19 and incident diabetes.

### 4.4 Implications and limitations

The pandemic of COVID-19 has placed a tremendous burden on humanity and might co-exist with us for many years. Our meta-analysis summarizes the existing evidence of the association between COVID-19 infection and the risk of new incident diabetes and shows that a history of COVID-19 is a risk factor for all-type diabetes. It suggests that the identification of high-risk groups of diabetes should cover patients with COVID-19, which is conducive to the early detection and management of diabetes. Vaccination is of critical importance for individuals to reduce the risks of adverse outcomes. More studies should be fostered to clarify the potential mechanisms underlying the COVID-related diabetes, given there might be a complex combination of pathophysiological processes behind the COVID-19 infection and new incident diabetes.

Meanwhile, this study has certain limitations. We only included cohort studies of which retrospective cohort studies are the majority. Though there is a broad and deep use of electronic databases based on validated definitions, it still cannot exclude the bias caused by misclassification, particularly for diabetes types. Moreover, the intervals between COVID-19 infection and diabetes diagnosis differ in studies, which might lead to high heterogeneity, and make it hard to discuss the risks of incident diabetes in different phases of COVID-19. Age stratification is diverse among included studies, so we did not pool related data.

## 5 Conclusions

Patients ever infected with COVID-19 had an elevated incidence and risk of new incident diabetes. However, more studies are necessary to specify the pathophysiological mechanisms underlying this association.

## Data Availability

The original contributions presented in the study are included in the article/**Supplementary Material**. Further inquiries can be directed to the corresponding author.
