# Supplementary material for: Association of COVID-19 infection and the risk of new incident diabetes: a systematic review and meta-analysis
Source: Front Endocrinol (Lausanne). 2024 Aug 26;15:1429848. doi: 10.3389/fendo.2024.1429848 (PMC11381376; doi:10.3389/fendo.2024.1429848)

**The retrieval strategies and retrieval results of each database are shown in Supplementary Tables 1-3**.

**Supplementary Table 1**: PubMed (Searched on 4^th^ February 2024).

| No. | Content | Result |
| --- | --- | --- |
| #1 | **Search: "COVID-19"[Mesh] Sort by: Most Recent** | 253,039（16:47:41） |
| #2 | **Search: ((((((((((2019 nCoV Infection*[Title/Abstract]) OR (SARS CoV 2 Infection*[Title/Abstract])) OR (2019 Novel Coronavirus*[Title/Abstract])) OR (COVID 19 Virus Infection*[Title/Abstract])) OR (COVID19[Title/Abstract])) OR (Coronavirus Disease 19[Title/Abstract])) OR (Severe Acute Respiratory Syndrome Coronavirus 2 Infection[Title/Abstract])) OR (COVID 19 Virus Disease*[Title/Abstract])) OR (2019 nCoV Disease*[Title/Abstract])) OR (SARS Coronavirus 2 Infection[Title/Abstract])) OR (COVID 19 Pandemic*[Title/Abstract]) Sort by: Most Recent** | 348,249  (16:51:20) |
| #3 | **Search: ("COVID-19"[Mesh]) OR (((((((((((2019 nCoV Infection*[Title/Abstract]) OR (SARS CoV 2 Infection*[Title/Abstract])) OR (2019 Novel Coronavirus*[Title/Abstract])) OR (COVID 19 Virus Infection*[Title/Abstract])) OR (COVID19[Title/Abstract])) OR (Coronavirus Disease 19[Title/Abstract])) OR (Severe Acute Respiratory Syndrome Coronavirus 2 Infection[Title/Abstract])) OR (COVID 19 Virus Disease*[Title/Abstract])) OR (2019 nCoV Disease*[Title/Abstract])) OR (SARS Coronavirus 2 Infection[Title/Abstract])) OR (COVID 19 Pandemic*[Title/Abstract])) Sort by: Most Recent** | 383,616（16:52:59） |
| #4 | **"Post-Acute COVID-19 Syndrome" [MeSH] Sort by: Most Recent** | 2,933  (16:54:54) |
| #5 | **((((((Post Acute COVID 19 Syndrome*[Title/Abstract]) OR (Long Haul COVID*[Title/Abstract])) OR (Post-Acute Sequelae*[Title/Abstract])) OR (Post COVID Condition*[Title/Abstract])) OR (LONG COVID[Title/Abstract])) OR (PASC Post Acute Sequelae*[Title/Abstract])) OR (Post Acute COVID-19 Syndrome[Title/Abstract]) Sort by: Most Recent** | 5,336  （16:57:10） |
| #6 | **Search: ("Post-Acute COVID-19 Syndrome" [MeSH]) OR (((((((Post Acute COVID 19 Syndrome*[Title/Abstract]) OR (Long Haul COVID*[Title/Abstract])) OR (Post-Acute Sequelae*[Title/Abstract])) OR (Post COVID Condition*[Title/Abstract])) OR (LONG COVID[Title/Abstract])) OR (PASC Post Acute Sequelae*[Title/Abstract])) OR (Post Acute COVID-19 Syndrome[Title/Abstract])) Sort by: Most Recent** | 5,727  (16:58:21) |
| #7 | **Search: Diabetes Mellitus[MeSH] Sort by: Most Recent** | 519,270  (16:59:54) |
| #8 | **Search: diabete*[Title/Abstract] Sort by: Most Recent** | 683,247  (17:00:56) |
| #9 | **Search: (Diabetes Mellitus[MeSH]) OR (diabete*[Title/Abstract]) Sort by: Most Recent** | 815,223  (17:02:12) |
| #10 | **Search: (("COVID-19"[Mesh]) OR (((((((((((2019 nCoV Infection*[Title/Abstract]) OR (SARS CoV 2 Infection*[Title/Abstract])) OR (2019 Novel Coronavirus*[Title/Abstract])) OR (COVID 19 Virus Infection*[Title/Abstract])) OR (COVID19[Title/Abstract])) OR (Coronavirus Disease 19[Title/Abstract])) OR (Severe Acute Respiratory Syndrome Coronavirus 2 Infection[Title/Abstract])) OR (COVID 19 Virus Disease*[Title/Abstract])) OR (2019 nCoV Disease*[Title/Abstract])) OR (SARS Coronavirus 2 Infection[Title/Abstract])) OR (COVID 19 Pandemic*[Title/Abstract]))) OR (("Post-Acute COVID-19 Syndrome" [MeSH]) OR (((((((Post Acute COVID 19 Syndrome*[Title/Abstract]) OR (Long Haul COVID*[Title/Abstract])) OR (Post-Acute Sequelae*[Title/Abstract])) OR (Post COVID Condition*[Title/Abstract])) OR (LONG COVID[Title/Abstract])) OR (PASC Post Acute Sequelae*[Title/Abstract])) OR (Post Acute COVID-19 Syndrome[Title/Abstract]))) Sort by: Most Recent** | 383,966  (17:04:17) |
| #11 | **Search: ((("COVID-19"[Mesh]) OR (((((((((((2019 nCoV Infection*[Title/Abstract]) OR (SARS CoV 2 Infection*[Title/Abstract])) OR (2019 Novel Coronavirus*[Title/Abstract])) OR (COVID 19 Virus Infection*[Title/Abstract])) OR (COVID19[Title/Abstract])) OR (Coronavirus Disease 19[Title/Abstract])) OR (Severe Acute Respiratory Syndrome Coronavirus 2 Infection[Title/Abstract])) OR (COVID 19 Virus Disease*[Title/Abstract])) OR (2019 nCoV Disease*[Title/Abstract])) OR (SARS Coronavirus 2 Infection[Title/Abstract])) OR (COVID 19 Pandemic*[Title/Abstract]))) OR (("Post-Acute COVID-19 Syndrome" [MeSH]) OR (((((((Post Acute COVID 19 Syndrome*[Title/Abstract]) OR (Long Haul COVID*[Title/Abstract])) OR (Post-Acute Sequelae*[Title/Abstract])) OR (Post COVID Condition*[Title/Abstract])) OR (LONG COVID[Title/Abstract])) OR (PASC Post Acute Sequelae*[Title/Abstract])) OR (Post Acute COVID-19 Syndrome[Title/Abstract])))) AND ((Diabetes Mellitus[MeSH]) OR (diabete*[Title/Abstract])) Sort by: Most Recent** | 11,895  (17:05:47) |
| #12 | Search: **risk[MeSH]** Sort by: **Most Recent** | [1,400,065](https://pubmed.ncbi.nlm.nih.gov/?term=risk[MeSH]&sort=date&size=200&ac=no)  (17:08:20) |
| #13 | Search: **risk[Title/Abstract]** Sort by: **Most Recent** | [2,855,533](https://pubmed.ncbi.nlm.nih.gov/?term=risk[Title/Abstract]&sort=date&size=200&ac=no)  (17:09:31) |
| #14 | Search: **(risk[MeSH]) OR (risk[Title/Abstract])** Sort by: **Most Recent** | [3,375,241](https://pubmed.ncbi.nlm.nih.gov/?term=(risk[MeSH])+OR+(risk[Title/Abstract])&sort=date&size=200&ac=no)  (17:10:12) |
| #15 | **Search: (((("COVID-19"[Mesh]) OR (((((((((((2019 nCoV Infection*[Title/Abstract]) OR (SARS CoV 2 Infection*[Title/Abstract])) OR (2019 Novel Coronavirus*[Title/Abstract])) OR (COVID 19 Virus Infection*[Title/Abstract])) OR (COVID19[Title/Abstract])) OR (Coronavirus Disease 19[Title/Abstract])) OR (Severe Acute Respiratory Syndrome Coronavirus 2 Infection[Title/Abstract])) OR (COVID 19 Virus Disease*[Title/Abstract])) OR (2019 nCoV Disease*[Title/Abstract])) OR (SARS Coronavirus 2 Infection[Title/Abstract])) OR (COVID 19 Pandemic*[Title/Abstract]))) OR (("Post-Acute COVID-19 Syndrome" [MeSH]) OR (((((((Post Acute COVID 19 Syndrome*[Title/Abstract]) OR (Long Haul COVID*[Title/Abstract])) OR (Post-Acute Sequelae*[Title/Abstract])) OR (Post COVID Condition*[Title/Abstract])) OR (LONG COVID[Title/Abstract])) OR (PASC Post Acute Sequelae*[Title/Abstract])) OR (Post Acute COVID-19 Syndrome[Title/Abstract])))) AND ((Diabetes Mellitus[MeSH]) OR (diabete*[Title/Abstract]))) AND ((risk[MeSH]) OR (risk[Title/Abstract])) Sort by: Most Recent** | 6042  (17:11:13) |

**Supplementary Table 2**: Embase (Searched on 4^th^ February 2024).

| No. | Content | Result |
| --- | --- | --- |
| #1 | **'coronavirus disease 2019'/exp** | 380,540 |
| #2 | **'2019 ncov infection*':ab,ti OR 'sars cov 2 infection*':ab,ti OR '2019 novel coronavirus*':ab,ti OR 'covid 19 virus infection*':ab,ti OR covid19:ab,ti OR 'coronavirus disease 19':ab,ti OR 'severe acute respiratory syndrome coronavirus 2 infection':ab,ti OR 'covid 19 virus disease':ab,ti OR '2019 ncov disease*':ab,ti OR 'sars coronavirus 2 infection':ab,ti OR 'covid 19 pandemic*':ab,ti** | 393,547 |
| #3 | **#1 OR #2** | 458,337 |
| #4 | **'long covid'/exp** | 6756 |
| #5 | **'post acute covid 19 syndrome*':ab,ti OR 'long haul covid*':ab,ti OR 'post-acute sequelae*':ab,ti OR 'post covid condition*':ab,ti OR 'long covid':ab,ti OR 'pasc post acute sequelae*':ab,ti OR 'post acute covid-19 syndrome':ab,ti** | 5798 |
| #6 | **#4 OR #5** | 8499 |
| #7 | **#3 OR #6** | 458,463 |
| #8 | **'diabetes mellitus'/exp** | 1,312,359 |
| #9 | **diabete*:ab,ti** | 1,038,221 |
| #10 | **#8 OR #9** | 1,475,753 |
| #11 | **'risk'/exp** | 3,174,670 |
| #12 | **risk:ab,ti** | 4,102,458 |
| #13 | **#11 OR #12** | 5,035,316 |
| #14 | **#7 AND #10 AND #13** | 14,829 |

**Supplementary Table 3**: Cochran Library (Searched on 4^th^ February 2024).

| No. | Content | Result |
| --- | --- | --- |
| #1 | MeSH descriptor: [COVID-19] explode all trees | 7441 |
| #2 | (2019 nCoV Infection*):ti,ab,kw OR (SARS CoV 2 Infection*):ti,ab,kw OR (2019 Novel Coronavirus*):ti,ab,kw OR (COVID 19 Virus Infection*):ti,ab,kw OR (COVID19):ti,ab,kw (Word variations have been searched) | 5418 |
| #3 | (Coronavirus Disease 19):ti,ab,kw OR (Severe Acute Respiratory Syndrome Coronavirus 2 Infection):ti,ab,kw OR (COVID 19 Virus Disease*):ti,ab,kw OR (2019 nCoV Disease*):ti,ab,kw OR (SARS Coronavirus 2 Infection):ti,ab,kw | 10311 |
| #4 | (COVID 19 Pandemic*):ti,ab,kw | 5975 |
| #5 | #1 OR #2 OR #3 OR #4 | 16053 |
| #6 | MeSH descriptor: [Post-Acute COVID-19 Syndrome] explode all trees | 178 |
| #8 | (PASC Post Acute Sequelae*):ti,ab,kw OR (Post Acute COVID-19 Syndrome):ti,ab,kw | 671 |
| #9 | #6 OR #7 OR #8 | 3043 |
| #10 | #5 OR #9 | 16669 |
| #11 | MeSH descriptor: [Diabetes Mellitus] explode all trees | 45441 |
| #12 | (diabete*):ti,ab,kw | 105440 |
| #13 | #11 OR #12 | 108636 |
| #14 | MeSH descriptor: [Risk] explode all trees | 56221 |
| #15 | (risk):ti,ab,kw | 298415 |
| #16 | #14 OR #15 | 302356 |
| #17 | #10 AND #13 AND #16 | 400 |

# Supplementary figure 1: Sensitivity analysis of the risk of new incident diabetes caused by COVID-19 infection.
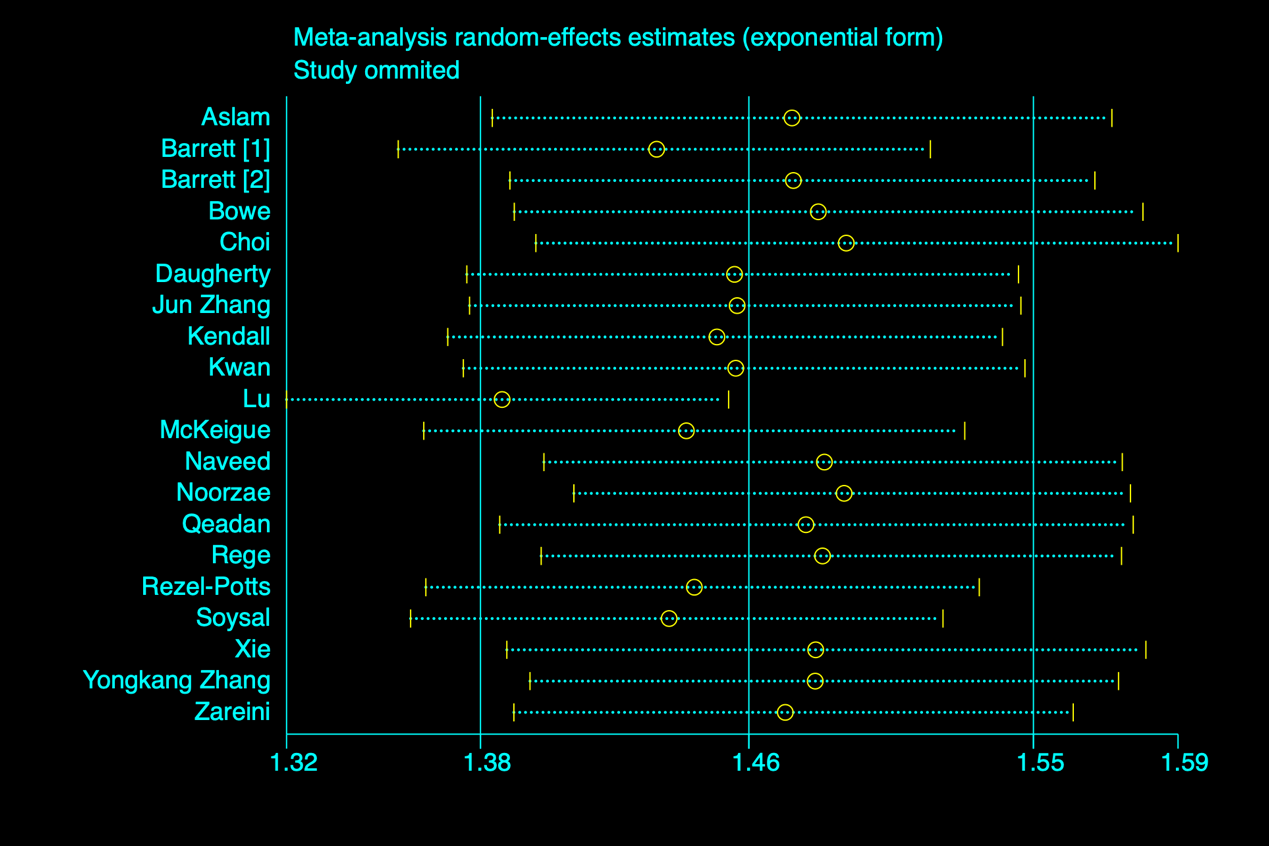

Supplement: Supplementary file 1 [file DataSheet1.doc]
